# Binary Alginate-Whey Protein Hydrogels for Antioxidant Encapsulation

**DOI:** 10.3390/antiox12061192

**Published:** 2023-05-31

**Authors:** Davide Pedrali, Alessio Scarafoni, Anna Giorgi, Vera Lavelli

**Affiliations:** 1 Department of Food, Environmental and Nutritional Sciences (DeFENS), University of Milan, Via Celoria 2, 20133 Milan, Italy; 2 Department of Agricultural and Environmental Sciences-Production, Landscape and Agroenergy (DiSAA), University of Milan, Via Celoria 2, 20133 Milan, Italy; anna.giorgi@unimi.it; 3 Centre of Applied Studies for the Sustainable Management and Protection of Mountain Areas (CRC Ge.S.Di.Mont.), University of Milan, 25048 Edolo, Italy

**Keywords:** alginate, whey, β-lactoglobulin, hydrogel, encapsulation, antioxidant, in vitro digestion

## Abstract

Encapsulation of antioxidants in hydrogels, i.e., three-dimensional networks that retain a significant fraction of water, is a strategy to increase their stability and bioaccessibility. In fact, low oxygen diffusivity in the viscous gelled phase decreases the rate of oxidation. Moreover, some hydrocolloids such as alginate and whey proteins provide a pH-dependent dissolution mechanism, allowing the retention of encapsulated compounds in the gastric environment and their release in the intestine, where they can be absorbed. This paper reviews the information on alginate-whey protein interactions and on the strategies to use binary mixtures of these polymers for antioxidant encapsulation. Results showed that alginate and whey proteins strongly interact, forming hydrogels that can be modulated by alginate molecular mass, mannuronic acid: guluronic acid ratio, pH, Ca^2+^ or transglutaminase addition. Hydrogels of alginate and whey proteins, in the forms of beads, microparticles, microcapsules, and nanocapsules, generally provide better encapsulation efficiency and release properties for antioxidants with respect to the hydrogel of alginate alone. The main challenges for future studies are to extend knowledge on the interactions among three components, namely alginate, whey proteins, and the encapsulated bioactive compounds, and to investigate the stability of these structures under food processing conditions. This knowledge will represent the rationale basis for the development of structures that can be tailored to specific food applications.

## 1. Introduction

Micro- and nanostructures such as molecular complexes, oil-in-water (O/W) emulsions, mycelles, and liposomes have been designed for encapsulation of both hydrophilic and lipophilic low molecular mass antioxidants in the liquid phase, in order to improve their solubility as well as stability and bioaccessibility [1,2,3]. In fact, food processing promotes antioxidant exposure to heat [4], light [5], and moisture [6], which results in oxidative degradation. Moreover, the bioaccessibility of antioxidants studied by in vitro simulation of mouth, stomach, and intestine digestion is generally low [7,8]. Besides encapsulation in liquid systems, research studies are also focusing on encapsulation strategies for antioxidants in hydrogels, which can be defined as three-dimensional networks that retain a significant fraction of water within their structure. In fact, encapsulation of antioxidants in hydrogels is expected to provide higher stability with respect to encapsulation in liquid systems, due to the diminished molecular diffusivity in a viscous gelled phase. For instance, the oxygen diffusion rate is 2.48 × 10^9^ m^2^ s^−1^ in water, while it decreases to 1.06 × 10^9^ m^2^ s^−1^ in miglyol, a model viscous oil [9], thus decreasing the rate of oxidative reactions. Furthermore, different food applications require a delivery device for antioxidants that prevents their release in the superior digestive tract and permits their release in the intestine at a predetermined rate. To this aim, various hydrogels are promising delivery systems since they provide a pH-dependent release mechanism.

Alginate is one of the most common food hydrocolloids. It is extracted primarily from brown algae such as *Laminaria* sp., *Durvillaea* sp., and *Sargassum* sp. Alginate is a linear anionic polysaccharide chain composed of 1,4-glycosidic bond-linked α-_L_-guluronic acid (G) residues and β-Dmannuronic acid (M), occurring as MM-, GG-, and MG-GM-block structures [10]. The molecular mass of alginate generally falls in the range of 32–400 kDa depending on the source, as well as on processing. For instance, alginate can be depolymerized effectively using high intensity ultrasounds (200 W, 24 kHz, 30 min at temperatures of 25 or 75 °C) and different amplitudes (50 or 100%) [11]. Alginate can form hydrogels in the presence of cations, which can shield the electrostatic repulsion among carboxyl groups through direct binding by ionic bonds. Alginate gelation processes with H^+^ or Ca^2+^ are the most common in food applications. The H^+^-type alginate gels can be formed when the pH of alginate solutions is below the pKa of the uronic acid residues, which are 3.65 and 3.38 for G and M, respectively, in 0.1 M NaCl [12]. Conversely, the Ca^2+^ gelation can be performed in a wide pH range, but the resulting gels have different properties. In particular, the Ca^2+^ alginate gel obtained at pH 3.8 (which is above but close to the pKa of alginate) has stronger chain interactions and hence a denser and interconnected microstructure than Ca^2+^ alginate gels obtained at a pH of 5.0 and 6.8 [13].

Due to the pH sensitivity of alginate, this polymer is used for controlled release of entrapped molecules in the intestine. The kinetic of release of encapsulated compounds is studied under in vitro digestion conditions. The mechanical properties of the hydrogels, such as the elastic modulus (Young’s modulus), have also been investigated at different stages of in vitro digestion, since they are closely associated to the release properties. For Ca^2+^ alginate gels, in simulated saliva, an increase in Young’s modulus is observed due to partial exchange of Ca^2+^ with Na^+^. In simulated gastric fluid (SGF), the Young’s modulus increases further and alginate gels shrink due to the protonation of free carboxylic groups and decreased repulsive forces between polymer chains. Conversely, in simulated intestinal fluid (SIF), the Young’s modulus decreases since alginate gels swell and break, due to the ion exchange between Ca^2+^ in gels and Na^+^ in digestive juice and increased repulsive forces between alginate monomers at high pH [14]. Consistent with the swelling behavior of alginate, the release of encapsulated compounds is generally faster in SIF than in SGF.

However, for hydrophilic antioxidants, the encapsulation efficiency in alginate hydrogel is critical. In fact, the alginate gel matrix is porous, which can lead to spontaneous diffusion and loss of hydrophilic antioxidants. Moreover, shrinkage of alginate microcapsules in SGF causes the gel syneresis along with the expulsion of water-soluble materials from the gel networks [15]. In addition, alginate is a hydrophilic polysaccharide and has poor emulsifying capacity because it lacks hydrophobic groups [16]. Hence, for lipophilic antioxidant encapsulation in alginate hydrogel, a lipid environment and a surfactant are necessary. Therefore, combining alginate hydrogels with a complementary polymer is a strategy to improve its encapsulation properties.

To this aim, β-lactoglobulin is a biopolymer that can extend alginate applications. It is the main component of whey, a byproduct of cheese-making, and it is used as purified protein or is present as the main protein in whey protein isolate (WPI, protein content 90%) or whey protein concentrate (WPC, protein content 50–80%) [17]. β-lactoglobulin is a globular protein of 162 amino acid residues, with a monomer molecular mass of 18.3 kDa, two disulfide bridges, and a free thiol group (SH). β-lactoglobulin displays intrinsic encapsulation ability for low molecular mass lipophilic compounds, primarily due to its conical central cavity (the calyx or β-barrel) that provides the main ligand binding site [18]. Moreover, both β-lactoglobulin and WPI can form hydrogels upon heating at 80 °C for approximately 30 min, which represents the ultimate result of a cascade of physicochemical events, including protein unfolding, aggregation, and gelation. Whey protein gel structure is stabilized by both covalent (disulfide) bonds and noncovalent protein–protein interactions, such as hydrophobic and ionic interactions and hydrogen bonding [19,20]. In salt-free solutions, opaque particulate gels composed of random aggregates occur at pH values near the isoelectric point (pI) of β-lactoglobulin (pH 4–6), whereas transparent fine-stranded gels are obtained by shifting the pH far from the pI [21]. Indeed, WPI hydrogels display pH-sensitive swelling behavior with minimum swelling ratio near the isoelectric point (pI) [20]. Ca^2+^-driven gelation of β-lactoglobulin or WPI can also be achieved through two consecutive steps: the preparation of a heat-denatured protein suspension, and chilling and addition of CaCl_2_ which enables cross-linking of proteins and thus promotes gelation. This latter process is called cold gelation because protein denaturation occurs before gelation and is preferred for encapsulation of heat-sensitive compounds [22].

Beside intrinsic encapsulation properties and gelling ability, WPI can act as a surfactant, because of its amphiphilic nature. Hence, WPI rapidly adsorbs to the emulsion interface where it self-aggregates and forms continuous and homogeneous membranes around the oil droplets through intermolecular β-sheets interactions. Then, gelation of these structures can be promoted by both Ca^2+^ or heat treatment [23].

To extend the properties that alginate and WPI/β-lactoglobulin display when used as single hydrocolloids, binary combinations of these polymers have been designed. This review analyzes the strategies to combine alginate and WPI/β-lactoglobulin with the aims to summarize the current knowledge regarding the nature of interaction between these polymers and to discuss the advantages of using both polymers for encapsulation of hydrophilic and lipophilic antioxidants. This will lead to identify future research needs to optimize food applications of antioxidants encapsulated in these hydrogels.

## 2. Materials and Methods

### Literature Study

The literature study was performed using two databases (Scopus and Web of Science). The search included the following keywords: “alginate” and “whey”. Papers reporting the development of an encapsulation technology for antioxidants were selected, while those related to the encapsulation of microorganisms, protein, and enzymes were not included in the focus of this review.

## 3. Results and Discussion

### 3.1. Alginate-Whey Protein/β-Lactoglobulin Interactions

The analysis of the ζ-potential of polymer solutions was applied to predict the interactions between alginate and WPI [24] or alginate and β-lactoglobulin [25] in the pH range 2–7. The ζ-potential of both the WPI solution and β-lactoglobulin solution changed from negative at pH 7.0 to positive at pH 2.0, with a point of zero charge around pH 4.7, which corresponds to protein pI. The ζ-potential of the alginate solution remained negative across the whole pH range studied, changing from strongly negative at pH 7 to slightly negative at pH 2 [24,25]. It would be expected that these biopolymers should be attracted to each other at pH values where they have opposite charges, but repel each other where they have the same charge, i.e., above pH 4.7. However, alginate and WPI/β-lactoglobulin were associated up to pH 5.5, which can be attributed to the binding of anionic groups on the alginate molecules to cationic patches on the surfaces of the protein molecules [24,25]. The interactions between alginate and WPI were then studied after Ca^2+^ addition to promote gelation. As a result, at pH values of 3.0, 5.0 or 7.0, the amount of protein retained in the hydrogel was found to increase from 11.6% at pH 7.0, to 19.1% at pH 5, to 58.6% at pH 3.0. Moreover, the release of protein in phosphate buffer occurred relatively slowly at pH 3.0, while it was rapid at pH 5.0 and 7.0. This trend was related to the strength of the electrostatic interactions between the protein and alginate molecules [24]. The pH-dependent complex formation between alginate and pure β-lactoglobulin was also studied by transmission electron microscopy, and maximum complex formation was found to occur at pH 4.2 [26]. The binding enthalpy at pH 4.2 was found to be −411 kJ/mol while entropy variation was −1.3 kJ/mol [26]. The exothermicity was attributed to electrostatic interactions between β-lactoglobulin and alginate, which surpasses the energy changes associated to the reduction in the number of hydrogen bonds with water, consequent to protein–alginate interaction.

In a further study, using hetero-nuclear single quantum coherence NMR spectroscopy, two different alginate binding sites were identified for monomeric β-lactoglobulin (isoform A) at pH 2.65; in contrast, only one site was observed at pH 4.0, where β-lactoglobulin occurs as a dimer, confirming that the alginate–protein network strongly depends on pH [27]. The stoichiometry of the complex and the apparent enthalpy variation also depend on G to M ratio. In fact, the β-lactoglobulin binding capacity is higher for alginate with higher M content [28]. For instance, for three alginate chains with the same average molecular mass of about 300 kDa and M/G ratios of 1.8, 1.1, and 0.6, the stoichiometry of the complexes at pH 4.0 was calculated by isothermal calorimetry, resulting 47.9, 43.4, and 28.3 mol β-lactoglobulin/mol alginate (6.8, 6.3, and 4.7 g of β-lactoglobulin per g of alginate), with enthalpy values of −83.4, −76.3, and −73.6 kJ/mol, respectively [28]. The effect of the molecular mass of alginate on the thermodynamic parameters of β-lactoglobulin—alginate complex was also studied considering a system made of binary mixtures of β-lactoglobulin and either low molecular mass alginate (40 kDa) or high molecular mass alginate (280 kDa) or an alginate trisaccharide. The dissociation constant Kd was approximately 10-fold higher for low molecular mass alginate-β-lactoglobulin complex than high molecular mass alginate-β-lactoglobulin complex and 3 orders of magnitude higher for an alginate trisaccharide-β-lactoglobulin complex, as determined by isothermal titration calorimetry. From this approach, it was found that the higher the molecular mass of alginate, the higher the enthalpy for binding with β-lactoglobulin [29].

Besides the occurrence of charge interactions between alginate and β-lactoglobulin, hydrophobic interactions can also be established. Raman spectroscopy analysis revealed that the interactions between WPI and alginate cause conformational changes in WPI; in particular, the ordered structures of α-helixes and β-sheets of WPI were strengthened at pH 4.0 and 5.0 [30,31]. In a further study, the effect of β-lactoglobulin cross-linking using transglutaminase on the binding with alginate was investigated. Under the reaction conditions chosen, cross-linked β-lactoglobulin molecular mass spanned 18 to >240 kDa, while alginate used for the study had a molecular mass of 139 kDa. Crosslinking of β-lactoglobulin changed the number of molecules of protein per molecule of alginate in the complex from 35 to 43. Moreover, there was a moderate increase in the apparent free energy from −42 to −34 kJ/mol and a sharp increase in entropy from 72 to 87 J/mol, as measured at pH 3.0, reflecting changes in the nature of interactions with respect to native β-lactoglobulin. This trend was explained with hypothesizing that hydrophobic interactions occurred among cross-linked β-lactoglobulin and alginate. Indeed, in alginate, the uronate ring side opposite of the carboxylic acid is hydrophobic and CD spectroscopy revealed that cross-linked β-lactoglobulin was structurally similar to heat-treated β-lactoglobulin, suggesting that the protein was unfolded. It was concluded that cross-linking of β-lactoglobulin or whey protein mixtures prior to interaction with alginate can expand the applications of the binary mixtures of these polymers [32].

Research so far performed has proven that strong interactions occur between alginate and WPI/β-lactoglobulin, which can be modulated by M:G ratio, alginate molecular mass, pH, Ca^2+^, and transaminase. This information has stimulated the development of binary matrices made of these polymers. Whey protein/β-lactoglobuin and alginate mixtures were studied for the production of edible coating and film. Interestingly, the β-lactoglobulin binding properties were maintained in the dry film and after film re-dissolution, which would allow the development of new carriers for food bioactive compounds [31]. The presence of β-lactoglobulin improved the oxygen barrier properties of the film [33] but decreased the tensile strength, and hence further studies are required before using these edible films for application in the food industry [31,33]. A variety of gelled structures made of alginate and WPI/β-lactoglobulin can be obtained, which are referred to with different denominations depending on the size and structure, such as beads (or spheres), capsules, microparticles, and nanocomplexes [33].

Beads or spheres are hydrogel matrices containing a dispersed bioactive compound, having a spherical shape and diameters in the range of millimeters. Depending on their polarity, the bioactive compounds dispersed in the hydrogel matrix of the beads can be directly solubilized in water and entrapped in the gel network or dissolved in lipid droplets, which in turns are entrapped in the gel network [34]. For hydrogels that are designed for possible applications into food products, acceptability is largely dependent on their perception within the mouth which, in turn, is affected by their size. Typically, particles larger than 50–100 μm can be detected as individual entities and give a gritty perception [35]. Hence, there is an interest in reducing the size of the hydrogels. Hydrogels with sizes in the range of 50–100 μm are called microparticles or microcapsules, depending on their inner structure. The microparticles generally refer to hydrogels containing a dispersed bioactive compound, which are irregular in shape [34]. The microcapsules are spheres that comprise a gelled membrane surrounding a liquid core, containing the bioactive compounds that can be either hydrophilic or hydrophobic. Nanoencapsulation provides some advantages with respect to microencapsulation due to the high area:volume ratio, resulting in high bioaccessibility of the encapsulated compounds.

In the following paragraphs, the strategies to encapsulate hydrophilic and lipophilic antioxidants in alginate-WPI/β-lactoglobulin hydrogels are described, which can lead to optimized delivery of these compounds in the food matrices.

### 3.2. Encapsulation of Hydrophilic Antioxidants in Alginate-Whey Protein/β-Lactoglobulin Hydrogels

#### 3.2.1. Encapsulation in Beads

**Figure 1:**
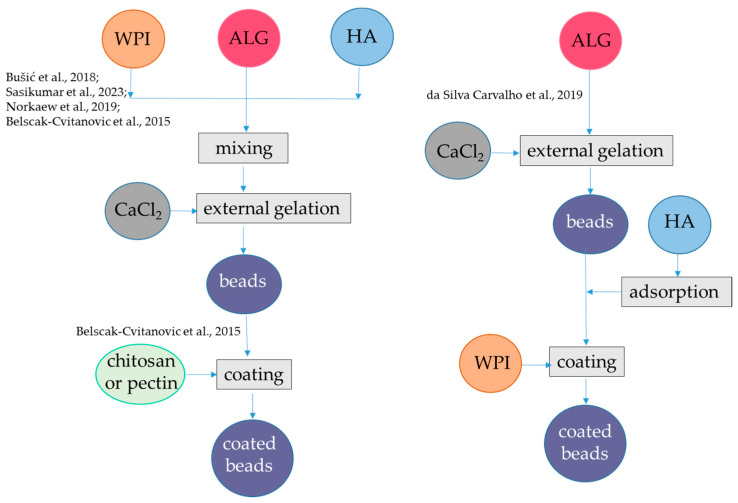
Steps to encapsulate hydrophilic antioxidants into alginate-whey protein beads. The procedures were applied to hydroxycinnamic acids from dandelion [39], flavanols from blood juice [41], anthocyanins from black rice [42], anthocyanins from jussara [43], and flavanols from green tea [44]. ALG, alginate; HA, hydrophilic antioxidants; WPI, whey protein isolate. The concentrations of polymers and antioxidants are indicated in Table 1.

Considering small hydrophilic antioxidant compounds, encapsulation in alginate hydrogel is challenging due to the porous nature of the network and the possible loss of these compounds for spontaneous diffusion and during gel shrinkage. Hence, the design of the network by combining alginate with another hydrocolloid such as WPI or β-lactoglobulin is crucial. In fact, at the low pH of SGF, carboxyl groups of alginate are protonized and hence the electrostatic repulsions among these groups lessened, favoring matrix shrinkage. In contrast, WPI chains (isoelectric point of 5.2) are positively charged, causing repulsive forces among matrix chains. Moreover, alginate slows pepsin attach to WPI. On the contrary, the ionic environment of SIF causes relaxation of the gel, making pancreatin attack easier on WPI and consequently favoring a fast release of encapsulated compounds [36]. As discussed before, alginate–protein hydrogel can be formed when these polymers have opposite charges but also when they have the same charge, in presence of Ca^2+^ [30,37]. The simplest protocol to encapsulate hydrophilic bioactive compounds includes preparation of a mixture of alginate, bioactive compound, and WPI. This solution is then dripped in CaCl_2_ for external gelation [38]. This protocol was applied to encapsulate the water-soluble extract of dandelion (*Taraxacum officinale*) leaf extract, which is composed of hydroxycinnamic acids, among which chlorogenic, caftaric, chicoric, and caffeic acids were identified [39]. The dandelion extract was added both to the polymer solution before gelation and to the CaCl_2_ solution (Figure 1, Table 1). The WPI–alginate beads so far obtained were compared with beads of pure alginate in terms of average dimensions, encapsulation efficiency, and in vitro digestion behavior. The average particle size of plain alginate beads was 2 mm, while that of beads with WPI was 1.76 mm [39]. Encapsulation efficiency for total phenolics was reported to be 82% in pure alginate and 93% in alginate added with WPI. On the other hand, the recovery of polyphenols was related to the amount added to the alginate/WPI solution before gelation, while the amount added to the CaCl_2_ solution was not considered. This is probably the reason for the observed high efficiency of encapsulation. The retained antioxidant capacity of dandelion leaf extract, determined as the ability to scavenge the ABTS (2,2′-azino-bis(3-ethylbenzothiazoline-6-sulfonic acid) radical was lower than the recovery of antioxidants, namely 61% in plain alginate and 81% in alginate added with WPI. FT-IR results confirmed the occurrence of interactions between dandelion polyphenols and the employed carriers, which may explain the decrease in antioxidant activity of the encapsulated compounds compared to the free extract, as the encapsulated bioactive compounds are involved in binding with the carriers. A slight decrease in bioactivity of phenolics encapsulated in alginate had already been observed and attributed to their interactions with the carrier [40]. The release profile of polyphenols in the beads formed by WPI and alginate showed a longer delay in SIG in the presence of two polymers than in the presence of alginate alone, but the exact percentages released were not reported. In the same study, carob or cocoa powders were also used as copolymers of alginate and found to provide less efficiency of encapsulation for phenolics but better delayed release in the digestive tract than WPI [39].

Using a similar approach, polyphenols from blood fruits (*Haematocarpus validus*), which are mainly composed by epigallocatechin, gallocatechin, gallic acid, catechin, 2-coumaric acid, and rutin, were encapsulated in alginate-β-lactoglobulin microbeads at pH 5.0, achieving an encapsulation yield of 84% (Figure 1, Table 1). This system led to a release of 14% of phenolics in SGF and 80% of phenolics in SIF. Moreover, the photo-oxidative stability of encapsulated phenolics was proven upon exposure to ultraviolet light (UV-C) at λ 253.7 for 90 min for sterilization [41]. Conversely, the same approach was not satisfactory to encapsulate anthocyanins. In fact, alginate-WPI beads containing water-soluble black rice (*Oryza sativa*) polyphenols extract as bioactive compounds were produced by external gelation (Figure 1, Table 1). The black rice extract was mainly composed by two anthocyanins, namely, cyanidin-3-*O*-glucoside and peonidin-3-*O*-glucoside. The particle size of the alginate-WPI microbeads was approximately 0.945 mm. The anthocyanin retention was low, i.e., 30%, which was attributed to the loss occurring during the gel formation of the alginate beads in the CaCl_2_ solution because these bioactive compounds are highly water-soluble. Conversely, in the same study it was found that almost 100% of anthocyanins were retained upon spray-drying with maltodextrin. The antioxidant activity showed the same trend as anthocyanin content, with higher value in the spray-dried maltodextin system than in aginate-WPI beads. The release of anthocyanins from the spray-dried maltodextrin was also better than the release from the alginate-WPI hydrogel. In fact, at the end of the in vitro gastrointestinal digestion, the alginate-WPI microbeads still had a dark purple color, indicating that a high portion of anthocyanins might have strong binding with the beads [42]. An alternative procedure was applied to encapsulate anthocyanins using negatively charged alginate and positively charged WPI (below the pI), which can form a bilayer (Figure 1, Table 1). Plain alginate beads with average diameters of about 1 mm were firstly produced at pH 6.5 and then immersed in a solution containing hydrophilic bioactive compounds, namely anthocyanins from jussara (*Euterpe edulis*) extract, which are mainly represented by cyanidin 3-*O*-rutinoside and cyanidin 3-*O*-glucoside. Due to the porous nature of the alginate hydrogel, the bioactive compounds could be adsorbed to the hydrogel structure. To prevent the diffusion of the bioactive compounds, coating with different polymers such as WPI, chitosan, and gelatin was applied at pH 3.5. Interestingly, the stability of encapsulated anthocyanins during refrigerated storage was investigated and results showed that the coating process using WPI was effective in protecting these compounds from degradation, with about 80% of retention after 1 month compared to 50% retention in the microbeads without WPI. The encapsulation efficiency was not reported, but it was shown that the antioxidant activity as measured with the ORAC (Oxygen Radical Absorbance Capacity) assay was higher for the uncoated alginate beads than for the coated alginate beads. One reason for this behavior could be that the coating polymer could interact with anthocyanin and decrease their antioxidant activity. However, the interaction of anthocyanin with the copolymer was not so strong to markedly delete their release in SGF. Indeed, the alginate beads released 76% anthocyanins, while the beads coated with chitosan, WPI, and gelatin released 73, 71, and 70%, of anthocyanins, respectively. In the SIF, the integrity of the beads was lost upon 20 min and the remaining anthocyanins were released [43].

The basic external gelation procedure can also be modified by introducing a coating step of alginate-WPI beads with other polymers [44]. Initially, a solution of alginate (molecular mass: 80–120 kDa) and WPI (soy protein, casein, bovine serum albumin, or hemp proteins were also used as an alternative to WPI) was blended in a green tea (*Camellia sinensis*) water extract and then the mixture was dripped into a CaCl_2_ solution containing the same green tea extract (Figure 1, Table 1). The composition of the green tea extract was not specified, while the ratio between flavan-3-ol monomers and polymers is relevant for the encapsulation efficiency in hydrogel, since the monomers are less retained in the matrix [45]. The hydrogel beads obtained were studied as such or coated with either a chitosan or a pectin solution prepared in CaCl_2_ and green tea extract at pH 2.65. The median diameter d(0.5) was in the range 0.573–1.124 mm, with the highest sizes for the pectin- and chitosan-coated beads. WPI addition to alginate increased the encapsulation efficiency for flavanols from 48 to 60%, and coating with pectin (but not with chitosan) further increased the encapsulation efficiency to 83%. As expected, the changes in the retained antioxidant activity followed the same pattern as that of flavan-3-ols. For the plain alginate-protein beads, the release of flavan 3-ols during in vitro digestion was complete and very rapid (10 min) in SGF, regardless of the type of proteins. However, coating wet beads pectin (but not with chitosan) enabled to prolong the release of flavan-3-ols in SGF, even if after 2 h of incubation in the SGF 50% of flavan-3-ols were released. A complete release of these compounds occurred in the SIF [44].

#### 3.2.2. Encapsulation in Microparticles

**Figure 2:**
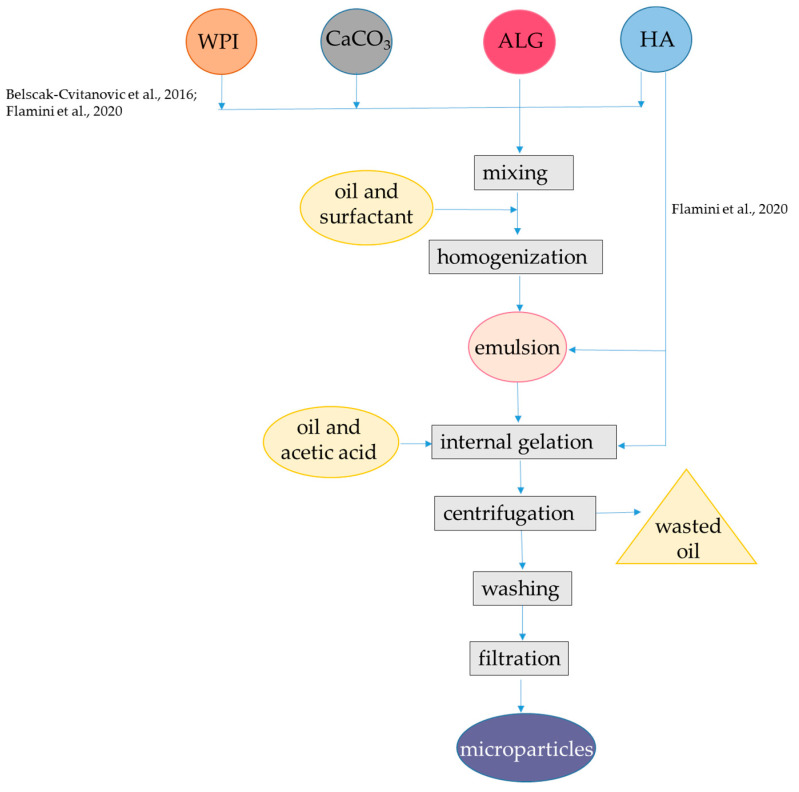
Steps to encapsulate hydrophilic antioxidants into alginate-whey protein microparticles. The procedures were applied to hydroxycinnamic acids from dandelion [48], oleuropein, hydroxytyrosol, and other phenolic compounds from olive oil leaf [49]. ALG, alginate; HA, hydrophilic antioxidants; WPI, whey protein isolate. The concentrations of polymers and antioxidants are indicated in Table 1.

The internal gelation technique was used to produce microparticles of alginate and WPI [46]. In contrast to particles formed via external gelation, the gel structure produced by internal gelation is more homogenous [47]. Internal gelation was applied to encapsulate the polyphenol extract of dandelion in alginate-WPI (Figure 2, Table 1), in comparison with the external gelation procedure applied using only alginate [48]. A water phase was prepared using alginate, WPI, and dandelion extract, mainly composed of hydroxycinnamic acids as indicated above. Then, CaCO_3_ was dispersed in the water phase. Sunflower oil containing Tween-80 as a surfactant and β-carotene was emulsified with the water phase. To promote internal gelation, sunflower oil containing glacial acetic acid was then added. Subsequently, the oil was removed by centrifugation and the microparticles were washed with ethanol. The microparticles obtained had average particle size of 0.3 mm, which was lower than that observed with the external gelation procedure (i.e., 2 mm).

Encapsulation efficiency for total hydroxycinnamic acids was about 80%, which was higher than that obtained by the external gelation procedure with alginate alone (i.e., 60%). Lower radical scavenging capacity as measured with the DPPH (2,2-diphenyl-1-picrylhydrazyl) radical was observed for internal/emulsion produced microparticles in comparison to plain alginate obtained by external/hydrophilic encapsulation, which might be due to the interaction between hydroxycinnamic acids and WPI. Indeed, hydrolysis of the microparticles and subsequent HPLC analysis coupled with FT-IR monitoring revealed that specific interactions occurred between hydroxycinnamic acids and the carrier polymers, especially in presence of WPI. Interestingly, WPI enabled β-carotene retention in the system, but the retention percentage was not reported. The high affinity of β-lactoglobulin for carotenoids, especially β-carotene had already been confirmed [50,51]. Results from the in vitro digestion showed that the release of hydroxycinnamic acids in SGF was lower than the release observed in SIF, while in the absence of WPI, the release of these compounds was not delayed [48]. The interactions with the carrier polymers can explain the slow release in SGF of encapsulated hydroxycinnamic acids. However, no information was provided on β-carotene release. The same procedure was also applied using different binary mixtures of polymers as the carrier materials for encapsulation, namely, hydroxypropyl methylcellulose in combination with alginate or pectin with either WPI or hydroxypropyl methylcellulose. Interestingly, the use of WPI in combination with alginate was the optimal carrier system for maximizing the encapsulation efficiencies of total polyphenols and hydroxycinnamic acids [48]. In one similar encapsulation procedure WPI, casein and pectin were compared as co-polymers of alginate to be used to encapsulate via internal gelation the phenolic-rich olive (*Olea europaea sativa*) leaf extract, which mainly consists of oleuropein and hydroxytyrosol [49]. In this latter study, Span 80 was used instead of Tween-80 and β-carotene was not added to the oil. In order to enhance the encapsulation efficiency, the same amount of extract used in the encapsulant solution was added in both the inversion and recovery media solutions. The mean particle size (D4,3) of the microparticles was in the range 48.2–65.1 μm. The low dimension of the microparticles could be related to the presence of the phenolic-rich olive leaves extract that is known to be surface active compounds [52,53,54]. The use of alginate in combination with WPI and casein improved the encapsulation efficiency from 20% up to 60%, while pectin increased encapsulation efficiency to 80%. This improvement was attributed to the development of interactions between alginate and WPI as well as between WPI and olive leaf phenolics. Interestingly, the alginate-WPI microparticles showed an enhanced antioxidant activity by the ABTS radical cation with respect to that expected based on their phenolic content, despite the occurrence of protein-polyphenols interactions, as confirmed by FT-IR spectra. Regarding the release of bioactive compounds, the microcapsules with WPI and casein showed higher swelling and release rates at pH 6.0 compared to pH 4.5, while those with pectin showed a fast release both at pH 4.5 and at pH 6.0 [49].

### 3.3. Encapsulation of Lipophilic Antioxidants in Alginate-Whey Protein/β-Lactoglobulin Hydrogels

#### 3.3.1. Encapsulation in Beads

**Figure 3:**
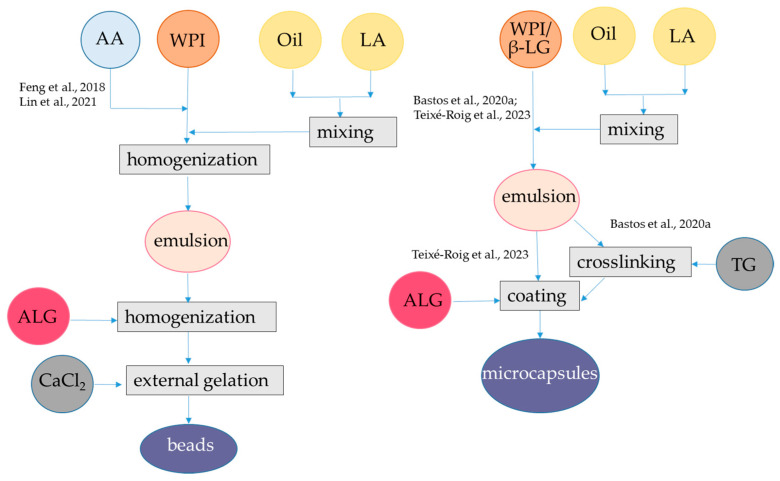
Steps to encapsulate lipophilic antioxidants into alginate-whey protein beads and microcapsules. The procedures were applied to α-tocopherol and resveratrol [58], lycopene [14], black pepper essential oil [62], and curcumin [64]. AA, amphiphilic antioxidants; ALG, alginate; β-LG, β-lactoglobulin; LA, lipophilic antioxidants; TG, transglutaminase; WPI, whey protein isolate. The concentrations of polymers and antioxidants are indicated in Table 1.

The encapsulation of lipophilic antioxidants in alginate-protein beads can be achieved using a carrier oil phase, which along with proteins acting as emulsifiers—forms small oil droplets that can be dispersed in the alginate hydrogel. When β-lactoglobulin is used as an emulsifier to stabilize oil droplets, coating of the droplets with alginate can be obtained from pH 3.0 to pH 6.0 at low ionic strengths, i.e., 100 mM NaCl. The alginate-coated WPI-oil droplets have better stability to flocculation. At pH 6 and 7, coating does not occur because of the strong electrostatic repulsion between the anionic alginate and anionic protein on the droplet surface [55,56]. However, in the presence of Ca^2+^, coating of β-lactoglobulin-stabilized emulsion can occur in a wide pH range, also above pH 6.0 where both polymers are negatively charged [57,58]. Following this latter approach, in one study, a primary emulsion was formed with sunflower oil containing α-tocopherol, resveratrol dissolved in ethanol, and denatured WPI at pH 7.0. Then, the emulsion was mixed with alginate at pH 7.0 and dripped into CaCl_2_ for gelation (Figure 3, Table 1). The oil droplet size ranged from 80 to 360 nm depending on the oil content (varying from 0.1% to 2%) and the WPI content (from 0.4 to 2%), while the gel beads carrying the oil droplets had diameters between 1.977 and 2.152 mm. The bioactive compounds showed different locations in the beads. In fact, α-tocopherol was dissolved in the oil phase while amphiphilic resveratrol was bound to WPI at the oil-water interface. Indeed, the interaction between resveratrol and β-lactoglobulin had already been proven [59,60]. The recovery of resveratrol in all WPI emulsions was >96% while recovery of α-tocopherol was about 85% when the contents of sunflower oil were 0.5% and 1% and >90% at higher oil contents. WPI content at 1% was effective in the protection of resveratrol during storage, with 60% remaining after 60 d at 25 °C. The stability of α-tocopherol was also improved, and its content was about 25% after 60 d [58]. The content of released resveratrol was about 91% and 97% after gastric digestion for 0.5 and 2 h, respectively. The release of α-tocopherol from all emulsion beads was basically close to or <10% after gastric digestion for 2 h and about 20% or less after gastrointestinal digestion for 6 h [58]. To encapsulate an oil phase containing lycopene extracted from tomato (*Lycopersicum eculentum*), alginate and WPI were used as carriers and gelation was performed at pH 7.0, in presence of Ca^2+^ as described above [14]. On the other hand, the amount of oil used to encapsulate lycopene emulsion was 10-fold higher than that used to encapsulate α-tocopherol emulsion (Figure 2, Table 1), which could be the reason for the higher bioaccessibility found for lycopene than for α-tocopherol, while the encapsulation efficiency for lycopene was not reported. The Authors found that the sizes of oil droplets delivering lycopene in gel beads (average size 3 mm) were significantly larger than the pores of hydrogel matrix, which are between 5 and 200 nm [61]. Therefore, lycopene and oil droplets were released from the gel beads due to structural degradation after swelling during the intestinal phase. Interestingly, the presence of WPI delayed the release of lycopene in the intestinal phase of digestion, probably due to a slower swelling rate of the beads. Lycopene bioaccessibility was found to be nearly 80% [14].

#### 3.3.2. Encapsulation in Microcapsules

Alginate-β-lactoglobulin microcapsules were obtained by using transglutaminase (Figure 3, Table 1). According to this procedure, β-lactoglobulin-alginate hydrogel with particle size in the range of 5 μm were produced at pH 4.5 using diluted polymer solution and transglutaminase as a crosslinking agent in place of Ca^2+^ to encapsulate black pepper (*Piper nigrum*) essential oil [62]. The black pepper essential oil contains different terpenes, among which the prevalent are β-caryophyllene, limonene, sabinene, β-pinene, and α-pinene. For encapsulation, a primary emulsion was formed with black pepper essential oil and β-lactoglobulin, then alginate was added, and the pH was adjusted to 4.5. Transglutaminase solution was added to induce crosslinking. Encapsulation efficiency of 80% was observed when β-lactoglobulin:alginate ratio was 17:1 and core:wall ratio was 2:1. FT-IR analysis revealed that the black pepper essential oil modified the tertiary structure of β-lactoglobulin, resulting from rearrangement of hydrophobic interactions, hydrogen, and ionic bonds. The microcapsule released about 30% of the encapsulated compounds under oral conditions while no release was observed under gastric conditions and up to 100% of the encapsulated compounds were released in the intestine, contributing 31% bioaccessibility for the essential oil [62]. Similar results were observed using lactoferrin in place of β-lactoglobulin [63].

Further studies evidenced that in the presence of 0.5% alginate, the gastric digestion of crosslinked WPI simulated in vitro is effectively reduced from 29% to 8%. The protection by alginate towards protein digestion was attributed to the charge interactions between polymers. Notably, digestion was also slowed in the intestinal phase even though the alginate protein particles were dissociating due to the pH of 7.0 of the SIF [65]. In another approach aimed at obtaining microcapsules to encapsulate curcumin, a carrier oil was used to dissolve curcumin and then added with WPI and alginate [64]. The particle size ranged between 0.487 and 2.251 µm, depending on alginate concentration. The addition of alginate did not affect the encapsulation efficiency, which was about 95%. When adding alginate, ζ-potential values of the microcapsules became more negative, and this is probably the reason for higher physical stability. On the other hand, with increasing alginate concentration from 0 to 1.5%, the bioaccessibility of curcumin decreased from 70% to 30% [64].

#### 3.3.3. Encapsulation in Nanoparticles

A nanoencapsulation strategy based on binary all material combination WPI-alginate was proposed using a carrier oil for lipophilic antioxidants. A primary emulsion was obtained with olive oil containing curcumin and WPI, at pH 7.0 by ultrasonication. Then, the pH of the primary emulsion was adjusted to 5, added with alginate, and sonicated further. Hence, the alginate layer was formed by electrostatic interaction with WPI, without Ca^2+^ addition. The average size of the primary emulsion droplets containing curcumin was 359 nm, whereas in the case of the secondary emulsion, it was increased to 841 nm. The encapsulation efficiency was 100%. In vitro digestion showed that in the gastric conditions, the emulsions remained stable, thereby not allowing the curcumin to be released from the emulsified droplets. Maximum releases of curcumin of almost 63 and 71% were attained after 2 h for the primary and secondary emulsion, respectively, and thereafter, it remained constant [66].

**Figure 4:**
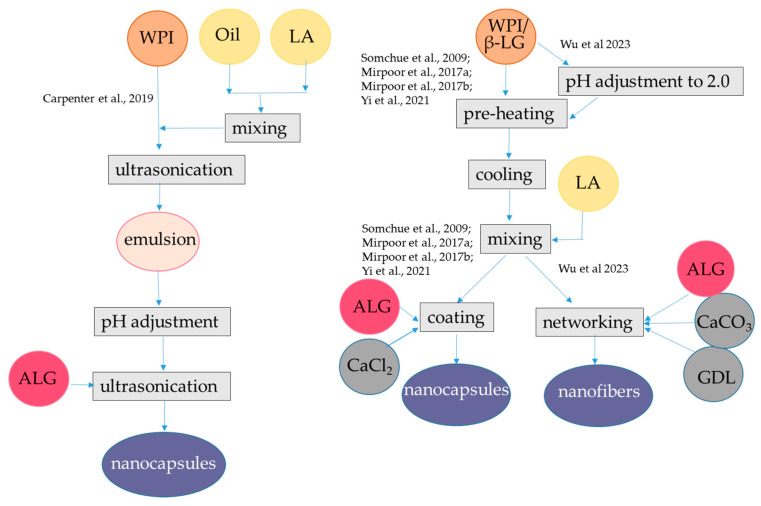
Steps to encapsulate lipophilic antioxidants into alginate-whey protein nanocapsules and nanofibers. The procedures were applied to α-tocopherol and resveratrol [69], quercetin [71], and curcumin [14,66,72,73]. ALG, alginate; β-LG, β-lactoglobulin; GDL, glucono δ-lactone; WPI, whey protein isolate. The concentrations of polymers and antioxidants are indicated in Table 1.

Alternatively, nanocapsules of β-lactoglobulin/WPI and alginate were also designed to encapsulate lipophilic antioxidants without the need of an oil phase, based on the affinity of β-lactoglobulin for small hydrophobic compounds. In one approach, β-lactoglobulin was added with caffeine and heated at 80 °C for 30 min, in order to allow heat-induced gelation. Then, the nanocomplexes obtained were coated with alginate in the presence of CaCl_2_. Additional layers of alginate coating were applied by repeating the procedure of alginate and CaCl_2_ addition up to four times. Encapsulation efficiency was not reported. This study demonstrated that the swelling and release behavior of the whey protein hydrogels can be changed easily with different layers of alginate coating. However, the release behavior under simulated digestion conditions was not investigated [20]. One point to notice is that bioactive compounds entrapped in integer nanoparticles may have decreased bioactivity. In fact, nanocomplexes of alginate, WPI, and thyme (*Thymus vulgaris*) oil with particle size of approximately 200 nm had decreased antimicrobial capacity, then free thyme oil [67,68]. Moreover, the heat-induced gelation procedure for encapsulation in WPI and alginate is only suitable for heat-stable bioactive compounds. In a further approach, these limitations were solved since β-lactoglobulin was first preheated at 80 °C for 30 min, cooled, and then was mixed with α-tocopherol as bioactive compound at 2:1 ratio. Then, the nanocomplexes formed were coated with alginate and CaCl_2_ (Figure 4, Table 1). Encapsulation efficiency was approximatively 20%. The positive effects of β-lactoglobulin and alginate interplay was observed in the release behavior under simulated digestion conditions, since evidence was provided that alginate-coated protein particles prolong the release of α-tocopherol till SIF conditions. In fact, the α-tocopherol retained in SGF was 55% and complete release occurred in SIF [69].

Self-assembly between alginate and whey protein (with no Ca^2+^ addition) can also be applied to create carrier nanostructures. A spontaneous association of oppositely charged alginate and β-lactoglobulin can occur at low concentrations (<4.5%); this phenomenon is also called complex coacervation [70]. This process was applied to encapsulate either quercetin or curcumin. These bioactive compounds were mixed with β-lactoglobulin at 1:1 ratio, which resulted in the formation of a nanocomplex. Then, alginate (molecular mass 200 kDa with M:G ratio of 0.6) was added at pH 4.0 in order to coat the nanocomplex (Figure 4, Table 1), obtaining an average particle size between 143 and 167 nm, depending on alginate concentration. The efficiency of encapsulation was above 90%. As observed previously [69], the release profile of the bioactive compounds from the nanoparticles under simulated digestion was improved due to the presence of alginate, since <3.5% release occurred during 6 h in SIF, while 77% release was observed during 12 h in SIF [71,72]. The physical stability of nanoparticles was investigated upon storage at pH 4.0 for 30 d at 25 °C, and upon HTST treatment at 75 °C for 30 s. Interesting, the β-lactoglobulin complexes were poorly stable upon both storage at pH 4.0 and heating, due to protein aggregation. Conversely, the physical stability of the nanoparticles made with both β-lactoglobulin and alginate was high, which was attributed to the anionic alginate shell that inhibited aggregation. Similarly, alginate shell provided a better chemical stability of quercetin and curcumin during storage at 45 °C for 18 d with respect to β-lactoglobulin alone, with about 50% of retention of both quercetin and curcumin [71,72]. In a further study, self-assembly of alginate and WPI was found to occur also at pH 5.0. In fact, the isoelectric point of WPI is 5.0 and hence the net charge is zero; there can still be some functional groups, including lysine residues, that are positively charged and can electrostatically interact with alginate. When pH was decreased to 4.5 or lower, strong electrostatic interactions occurred between WPI and alginate, leading to the formation of large insoluble aggregates. Conversely, above pH 5.5, both WI and alginate were negatively charged, and no electrostatic absorption occurred. Hence, at pH 5.0, alginate–WPI nanoparticles had an average size of 268 nm and were able to absorb curcumin added in ethanol. It was supposed that hydrophobic interactions were the main driving forces to promote curcumin binding with WPI, with encapsulation efficiency up to 85%. The WPI–alginate nanocomplex proved to be physically stable in high sucrose and NaCl concentration, and also at 90 °C up to 120 min [73].

In a different approach, WPI-alginate nanofibers were produced to encapsulate curcumin [74]. To obtain nanofibers, WPI was denatured by heating to 80 °C, at pH 2.0. Under these conditions, the native spherical protein was converted into a partially folded intermediate form containing β-sheets. Then, oligomers formed along the direction perpendicular to the β-sheets, forming protein nanofibers [75,76]. After formation of WPI nanofibers, the pH was adjusted to 6.5 and curcumin dissolved in ethanol was added. To form the binary microfibers, alginate, gluconate δ-lactone, and CaCO_3_ were finally added.

**Table 1:** Formulation of alginate-WPI/β-lactoglobulin hydrogels to encapsulate hydrophilic and lipophilic antioxidants *.

Structure-Wall Materials	Crosslinker	Core	Reference
Beads ALG 2.4%; WPI 4%	CaCl_2_ 2 or 3%	hydroxycinnamic acids extract from dandelion in water 1.2%	[39]
Beads: ALG 1.5%; β-LG 1%	CaCl_2_ 4%	phenolics from blood fruit extract in 80% aqueous ethanol 10%	[41]
Beads: ALG 0.5%; WPI 0.5%	CaCl_2_ 22%	anthocyanin extract from black rice in ethanol 0.25%	[42]
Beads: ALG 2%; WPC 1%	CaCl_2_ 2%	anthocyanin extract from jussara in water (n.p.)	[43]
Beads: ALG 1.28–1.6%; WPI 2%	CaCl_2_ 2%	flavanol extract from green tea in water (n.p.)	[44]
Microparticles: ALG 1.6%; WPI 2%	CaCO_3_ 0.5% acetic acid 5% in SO	hydroxycinnamic acids extract from dandelion in water (n.p.)	[48]
Microparticles: ALG 2%; WPI 2%	Ca-citrate 2% acetic acid 1.25% in SO	phenolics from olive oil leaf extracted in water 0.5%	[49]
Beads: ALG 1.4%; WPI 0.1–1%	CaCl_2_ 22%	SO 0.1–2%, α-tocopherol 1% in SO resveratrol in ethanol (n.p)	[58]
Beads: ALG 0.4%; WPI 2%	CaCl_2_ 2%	SO% 10% lycopene from tomato 0.015% in SO	[14]
Microcapsules: ALG + β-LG 0.45–1.8%	TG 0.25%	terpens of black pepper essential oil 0.45–1.8%	[62]
Microcapsules: ALG 0–1.5%; WPI 0–1.5%	-	CO 5% curcumin 0.1% in CO	[64]
Nanocapsules: ALG 0.2%; WPI 0.44%	-	OO 4.9% curcumin 0.022% in OO	[66]
Nanocapsules: ALG n.p; β-LG 0.5–2%	CaCl_2_ 1.1–11%	α-tocopherol 0.4–7%	[69]
Nanocapsules: ALG 0.05 or 0.019%; β-LG 0.025%	-	curcumin 0.0005%	[71]
Nanocapsules: ALG 0.05 or 0.019%; β-LG 0.025%	-	quercetin 0.0004%	[72]
Nanocapsules: ALG 0.16–0.05%; WPI 0.83–0.5%	-	curcumin 0.01%	[73]
Nanofibers ALG 1%; WPI 1–6%	CaCO_3_ 0.1% GDL 28.14 mM	curcumin 0.125%	[74]

* ALG, alginate; β-LG, β-lactoglobulin; CO, corn oil; n.p., concentration not provided; GDL, glucono δ-lactone; OO, olive oil; SO, sunflower oil; TG, transglutaminase; WPI, whey protein isolate; WPC, whey protein concentrate.

At this step, the hydrolysis of gluconate δ-lactone causes a slow release of Ca^2+^ from CaCO_3_, which then promotes the formation of a double network hydrogel between alginate and WPI [74]. The encapsulation efficiency was 91.6%. However, the release during in vitro digestion was not studied.

## 4. Conclusions

Interactions between alginate and WPI/β-lactoglobulin have been proven, which can be modulated by M:G ratio, protein denaturation, pH, and crosslinking with Ca^2+^ or transglutaminase. Interestingly, binary hydrogels can be formed both in a pH range where electrostatic forces occur due to opposite charge of these polymers and in a pH range where the two polymers are negatively charged, favored by the presence of Ca^2+^ ions.

Hydrophilic antioxidants can be encapsulated in alginate and WPI/β-lactoglobulin beads or microcapsules. In general, for hydrophilic antioxidants, the binary hydrogels improve the encapsulation efficiency with respect to the alginate hydrogel alone, probably due to the interactions between WPI/β-lactoglobulin and alginate, which allow the formation of a compact network. A delayed release in SIF from the beads and microparticles was observed for some hydrophilic antioxidant compounds.

Lipophilic antioxidants can be dissolved into an oil phase, emulsified by WPI/β-lactoglobulin and then coated with alginate in the presence of Ca^2+^ or transglutaminase in the form of beads or microcapsules. This approach leads to high encapsulation efficiency, but the release in SGF and SIF is low unless a high amount of oil (10%) is present. Alternatively, lipophilic compounds can be directly loaded into β-lactoglobulin followed by coating with alginate, without an oily phase, which leads to nanocapsules with high encapsulation efficiency and high release in SIF.

One point to notice is that in most of the studies so far performed, no information is reported regarding relevant factors such as alginate molecular mass, M:G ratio, and pH of gelation, which would be useful to correlate with the observed encapsulation efficiency and release properties. Additionally, for both hydrophilic and lipophilic bioactive compounds, interactions with the carrier polymers can cause a decreased antioxidant activity.

In this context, the main challenge for the future studies is to extend knowledge on the interactions among three components, namely alginate, WPI, and the encapsulated bioactive compound. This will represent the rationale basis for the development of structures that can be tailored to specific food applications. Moreover, knowledge on the physical and chemical stability of the structures under different conditions relevant to food processing and storage would help a better design of applications.
